# Supplementary material for: Allosteric activation unveils protein-mass modulation of ATP phosphoribosyltransferase product release
Source: Commun Chem. 2024 Apr 6;7:77. doi: 10.1038/s42004-024-01165-8 (PMC10998830; doi:10.1038/s42004-024-01165-8)
Supplement: Supplementary file 2 — Supplementary Information [file 42004_2024_1165_MOESM2_ESM.pdf]

Supplementary information

Allosteric activation unveils protein-mass modulation of ATP phosphoribosyltransferase product release

Benjamin J. Read[1], John B. O. Mitchell[2], and Rafael G. da Silva[1,*]

[1]School of Biology, Biomedical Sciences Research Complex, University of St Andrews, St Andrews, KY16 9ST, UK.

[2]EaStCHEM School of Chemistry, Biomedical Sciences Research Complex, University of St Andrews, St Andrews, KY16 9ST, UK.

*To whom correspondence should be addressed: Rafael G. da Silva, email: rgds@st-andrews.ac.uk.

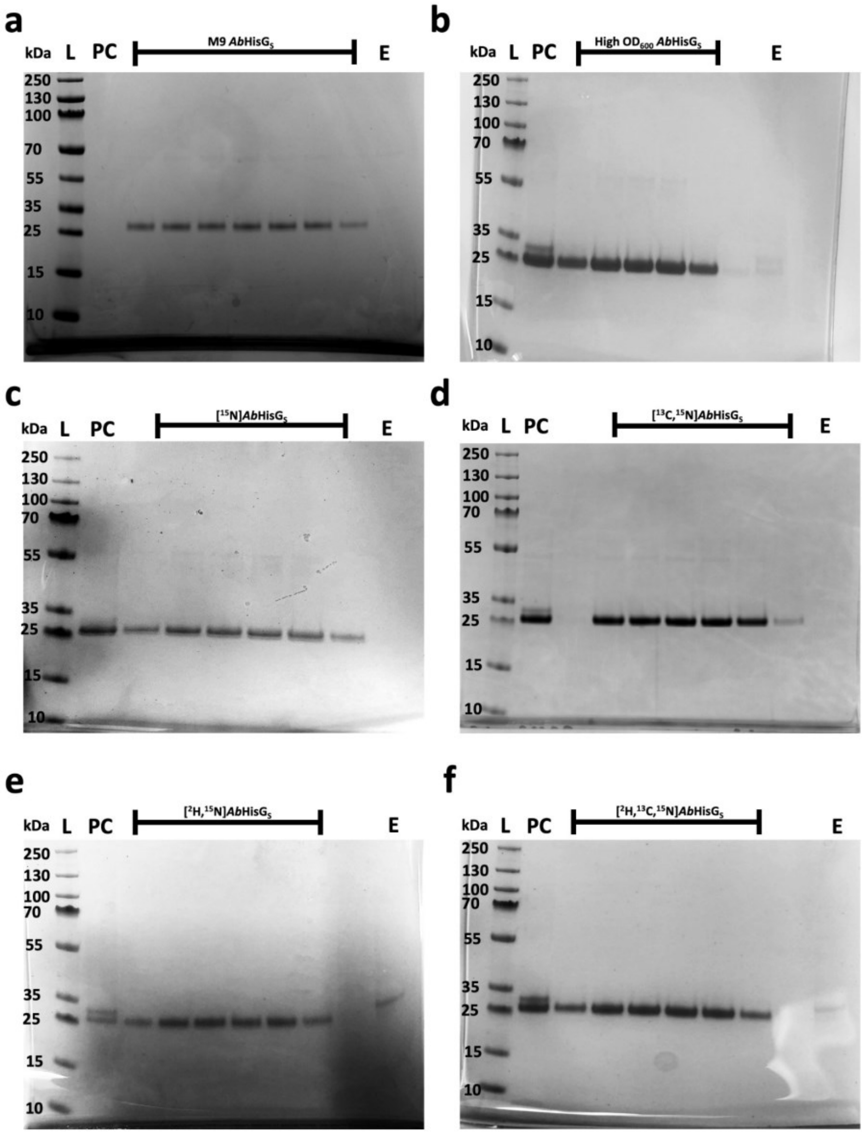

Supplementary Figure 1 SDS-PAGE analysis of purified *Ab*HisG$_S$ with distinct isotope labelling patterns eluted from the HisTrap FF column in the second chromatography. **a** Unlabelled *Ab*HisG$_S$ expressed in M9. **b** Unlabelled *Ab*HisG$_S$ expressed in M9 via the high cell density protocol. **c** [^{15}N]*Ab*HisG$_S$. **d** [^{13}C,^{15}N]*Ab*HisG$_S$. **e** [^{2}H,^{15}N]*Ab*HisG$_S$. **f** [^{2}H,^{13}C,^{15}N]*Ab*HisG$_S$. Lanes are as follows: L is the MW marker; PC is the pre-column sample; the top bar encompasses the flowthrough, which was pooled; E is the elution, which was discarded. The MW marker is the PageRuler Plus Prestained. Images **a** – **f** are representative of one batch of the respective *Ab*HisG$_S$ preparation;

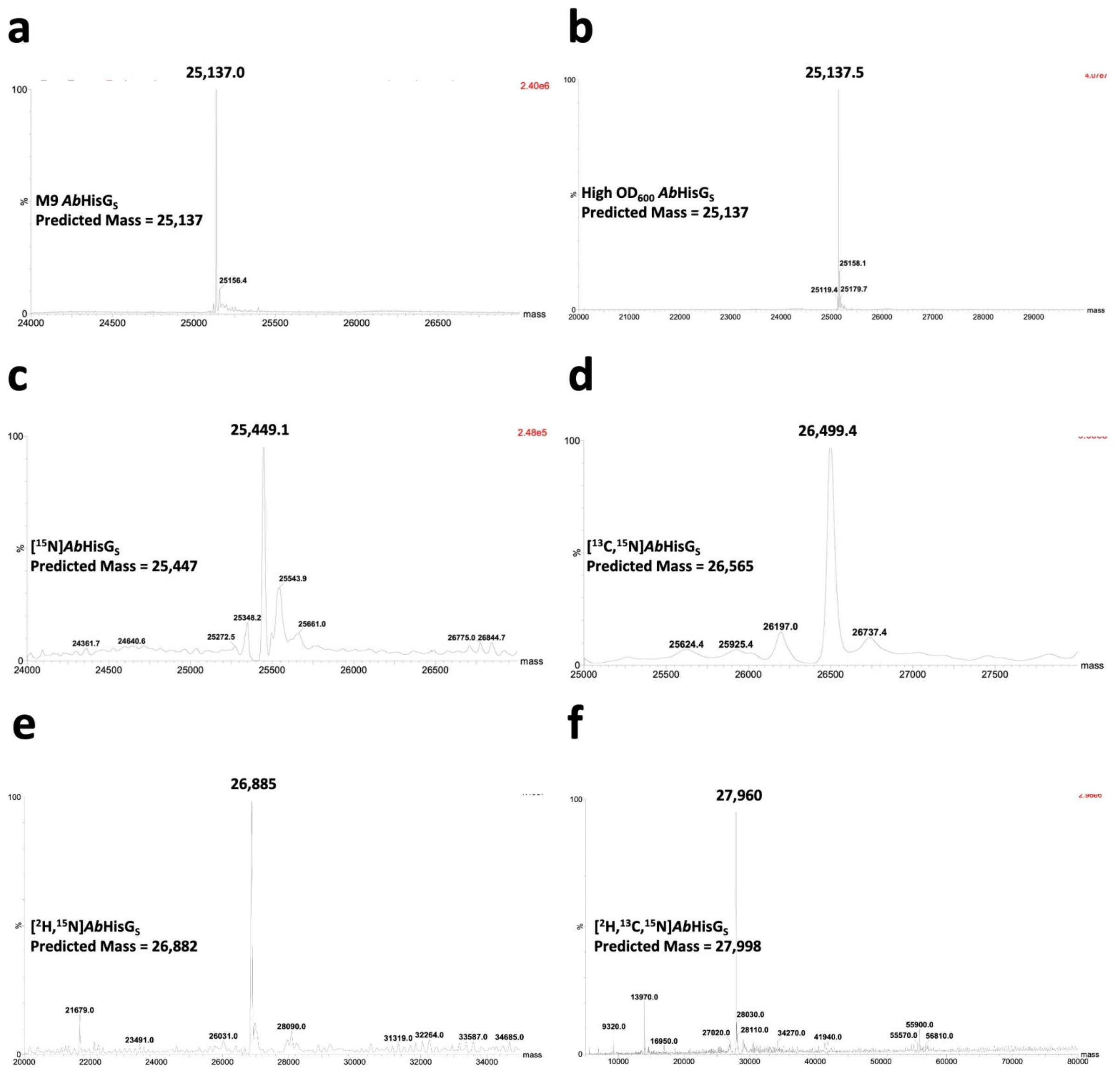

Supplementary Figure 2 ESI/TOF-MS analysis of *Ab*HisG$_S$ with distinct isotope labelling patterns. **a** Unlabelled *Ab*HisG$_S$ expressed in M9. **b** Unlabelled *Ab*HisG$_S$ expressed in M9 via the high cell density protocol. **c** [^{15}N]*Ab*HisG$_S$. **d** [^{13}C,^{15}N]*Ab*HisG$_S$. **e** [^{2}H,^{15}N]*Ab*HisG$_S$. **f** [^{2}H,^{13}C,^{15}N]*Ab*HisG$_S$.

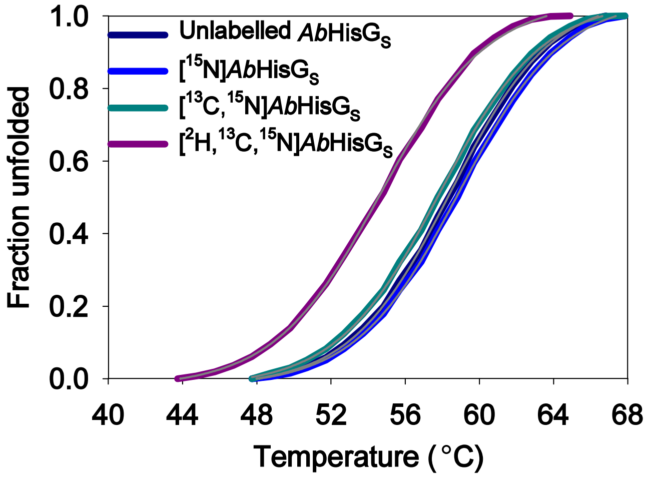

Supplementary Figure 3 DSF-based thermal denaturation of *Ab*HisG$_S$ and its isotope-labelled versions. Traces are averages of three independent measurements. Lines of best fit to equation (1) are in grey. T_ms (mean ± fitting error) are 58.49 ± 0.03 °C, 58.90 ± 0.04 °C, 57.84 ± 0.05 °C, and 54.67 ± 0.05 °C for unlabelled *Ab*HisG$_S$ (M9 high cell density), [^{15}N]*Ab*HisG$_S$, [^{13}C,^{15}N]*Ab*HisG$_S$, and [^{2}H,^{13}C,^{15}N]*Ab*HisG$_S$, respectively.

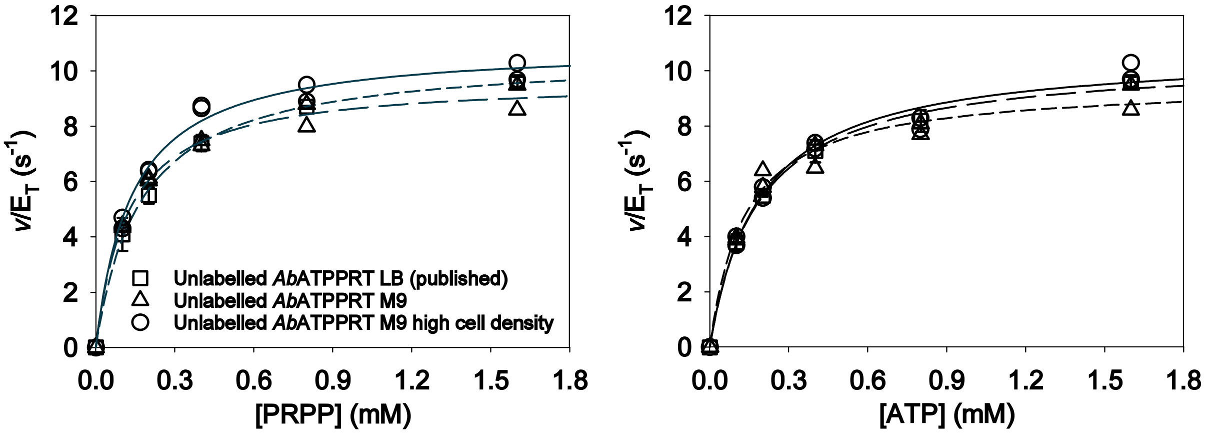

Supplementary Figure 4 Substrate saturation curves for unlabelled *Ab*ATPPRT with *Ab*HisG$_S$ purified from cultures grown in M9 and M9 with high cell density compared with previously published data (reproduced here) for unlabelled *Ab*ATPPRT with *Ab*HisG$_S$ purified from cultures grown in LB. For the M9 and M9 high cell density curves, all data points are shown for two independent measurements, except for the previously published LB curves, where data are the mean of two independent measurements. Lines are best fit of the data to equation (2): solid line for M9 high cell density curves, long-dashed line for M9 curves, and short-dashed line for previously published LB curves.

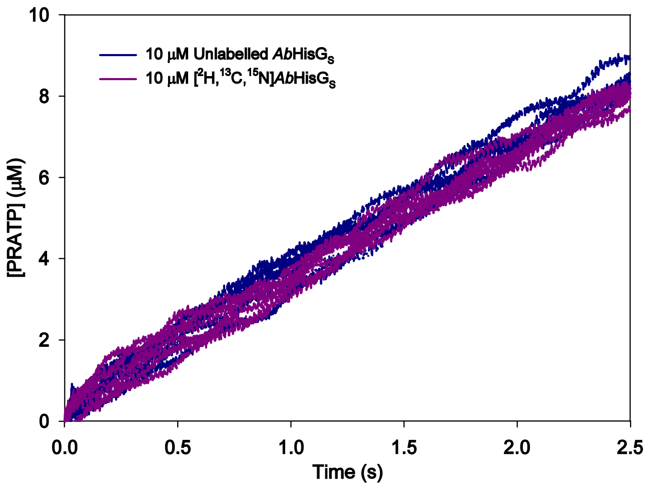

Supplementary Figure 5 Rapid kinetics of PRATP formation at 25 °C by unlabelled *Ab*HisG$_S$ and [^{2}H,^{13}C,^{15}N]*Ab*HisG$_S$ under multiple-turnover conditions. All traces are shown for eight replicates with each isotopologue.

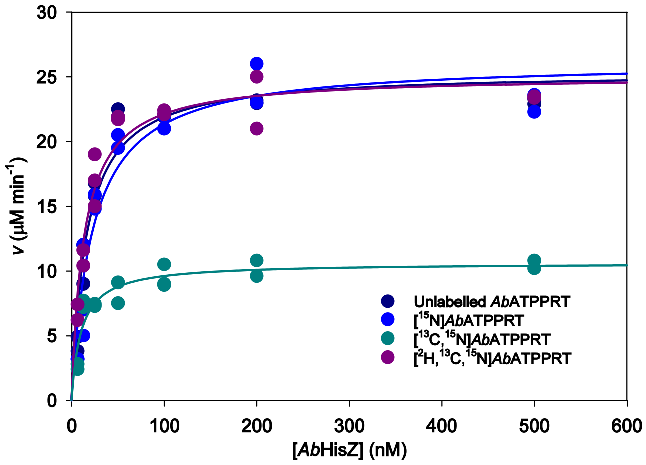

Supplementary Figure 6 Dependence of the rate of reaction catalysed by *Ab*HisGS isotopologues at 25 °C on the concentration of *Ab*HisZ. All data points are shown. Two independent measurements were carried out, except for [^{15}N]*Ab*HisGs with 12.5 nM *Ab*HisZ, [^{13}C,^{15}N]*Ab*HisGs with 100 nM *Ab*HisZ, and [^{2}H,^{13}C,^{15}N]*Ab*HisGs with 25 nM *Ab*HisZ, where three independent measurements were performed. Best fits of the data to equation (3) are shown as solid line. The concentration of all *Ab*HisGs isotopologues was 0.04 µM, except for [^{13}C,^{15}N]*Ab*HisGs, whose concentration was 0.02 µM.

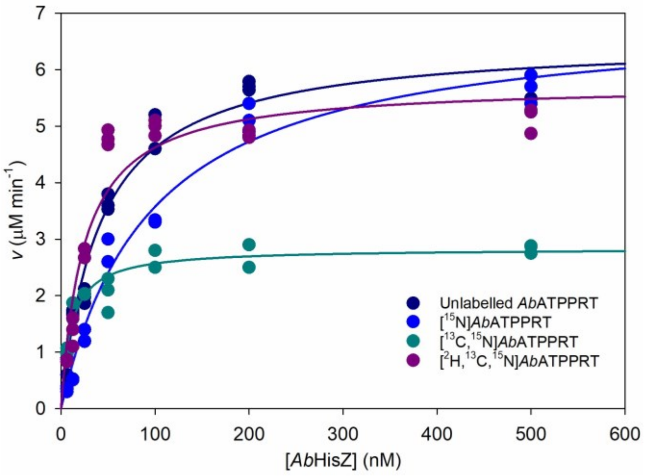

Supplementary Figure 7 Dependence of the rate of reaction catalysed by *Ab*HisG$_S$ isotopologues at 5 °C on the concentration of *Ab*HisZ. All data points are shown. Three independent measurements were carried out, except for [^{15}N]*Ab*HisG$_S$ with 12.5 nM and 50 nM *Ab*HisZ and [^{13}C,^{15}N]*Ab*HisG$_S$ with 6.5 nM and 200 nM *Ab*HisZ, where two independent measurements were performed. Best fits of the data to equation (3) are shown as solid line. The concentration of all *Ab*HisG$_S$ isotopologues was 0.04 μM, except for [^{13}C,^{15}N]*Ab*HisG$_S$, whose concentration was 0.02 μM.

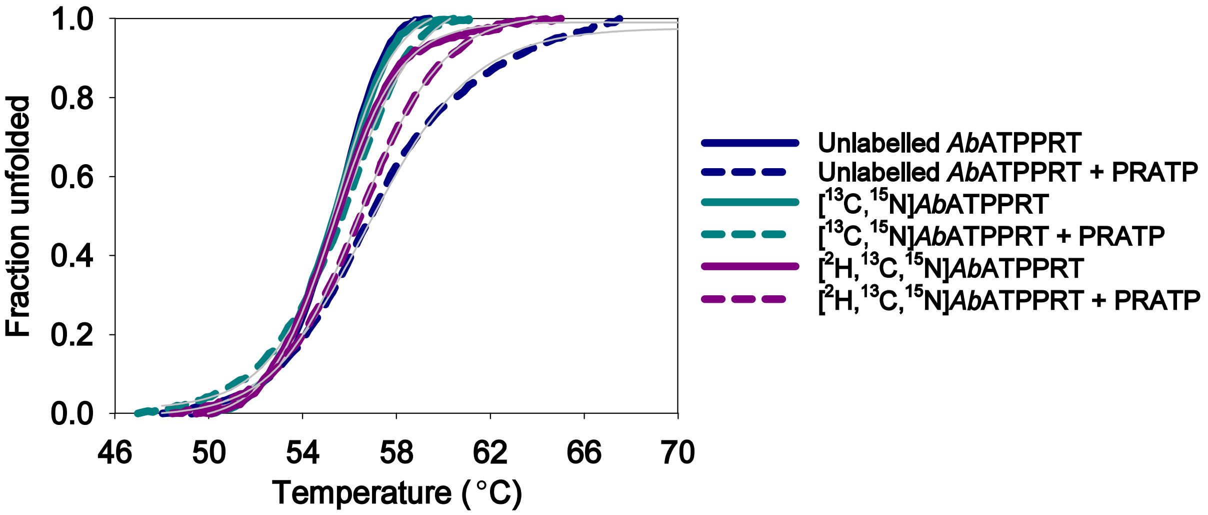

Supplementary Figure 8 DSF-based thermal denaturation of *Ab*ATPPRT isotopologues in the presence and absence of PRATP. Traces are averages of three independent measurements. Lines of best fit to equation (1) are in grey.

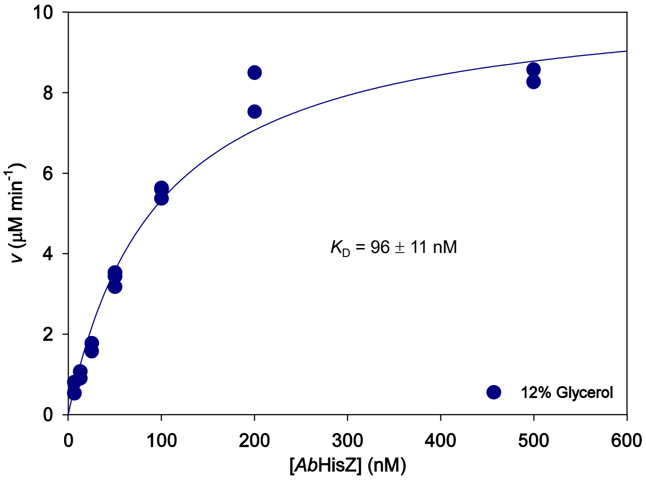

Supplementary Figure 9 Dependence of the rate of reaction catalysed by *Ab*HisG$_S$ at 5 °C on the concentration of *Ab*HisZ in the presence of 12% glycerol (v/v). All data points are shown. Three independent measurements were carried out, except at 6.25 nM, 12.5 nM, and 200 nM *Ab*HisZ, where two independent measurements were performed. Best fit of the data to equation (3) is shown as solid line.

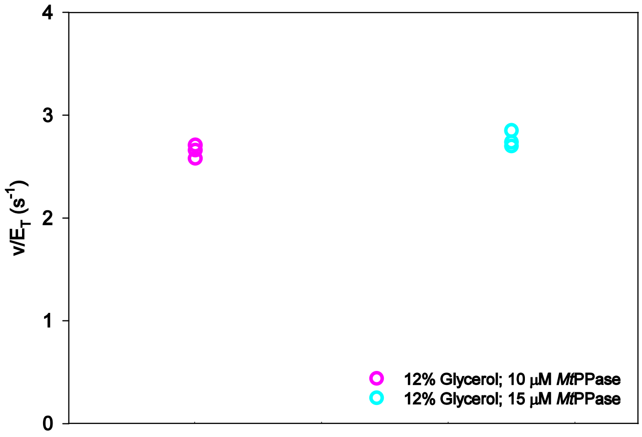

Supplementary Figure 10 Rate of reaction catalysed by *Ab*ATPPRT at 5 °C in the presence of 12% glycerol (v/v) and different concentrations of *Mt*PPase. All data points are shown. Three independent measurements were carried out.

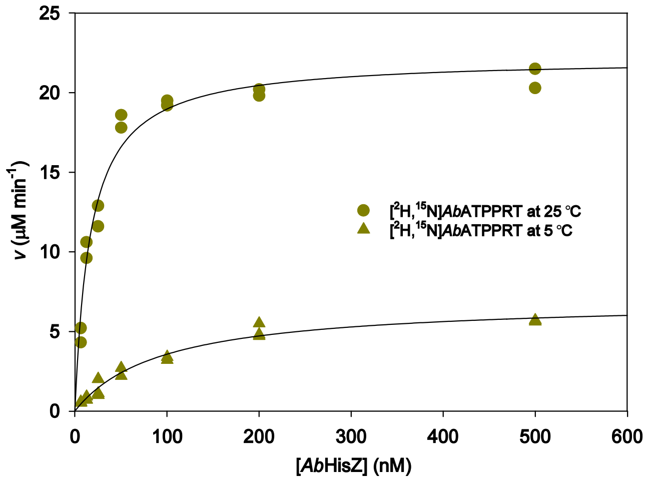

Supplementary Figure 11 Dependence of the rate of reaction catalysed by [^{2}H,^{15}N]*Ab*HisG$_S$ at 25 °C and 5 °C on the concentration of *Ab*HisZ. All data points are shown. Two independent measurements were carried out, except at 6.25 nM, 25 nM, and 200 nM *Ab*HisZ at 5 °C, where three independent measurements were performed. Best fits of the data to equation (3) are shown as solid lines.

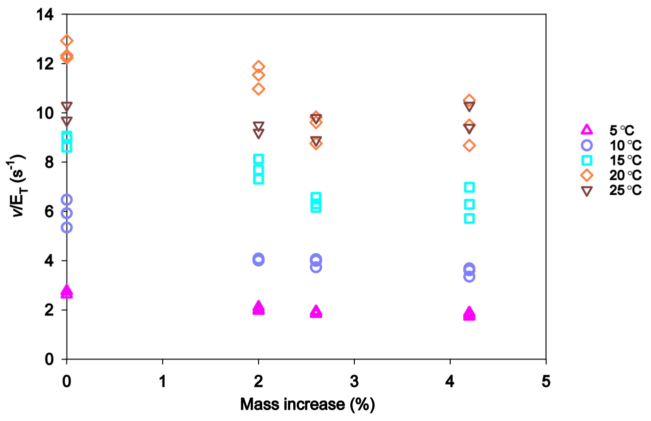

Supplementary Figure 12 Activity of *Ab*ATPPRT isotopologues at different temperatures. PRPP and ATP concentrations are those saturating at 5 °C and 25 °C. All data points are shown for three independent measurements, except at 25 °C, where all data points are shown for two independent measurements.

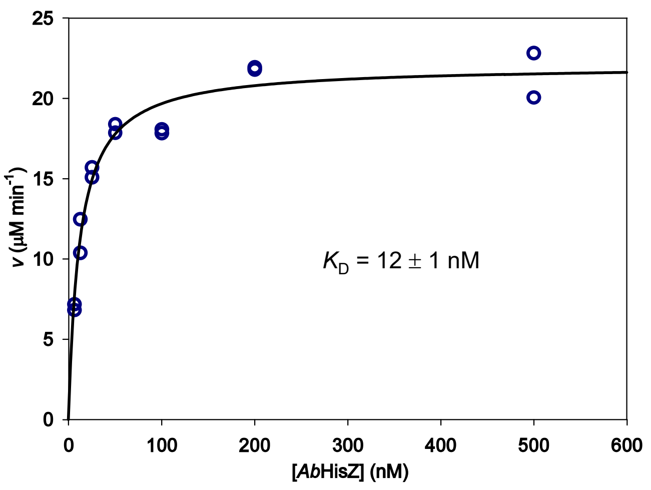

Supplementary Figure 13 Dependence of the rate of reaction catalysed by unlabelled *Ab*HisG$_S$ at 35 °C on the concentration of *Ab*HisZ. All data points are shown for two independent measurements. Best fit of the data to equation (3) is shown as a solid line.

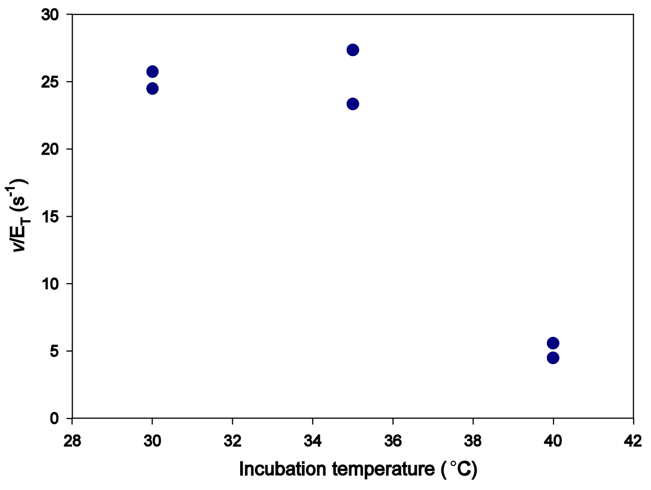

Supplementary Figure 14 Temperature stability of *Ab*ATPPRT activity. AbATPPRT was incubated at different temperatures for 10 min before activity assay at 30 °C. All data points are shown for two independent measurements.

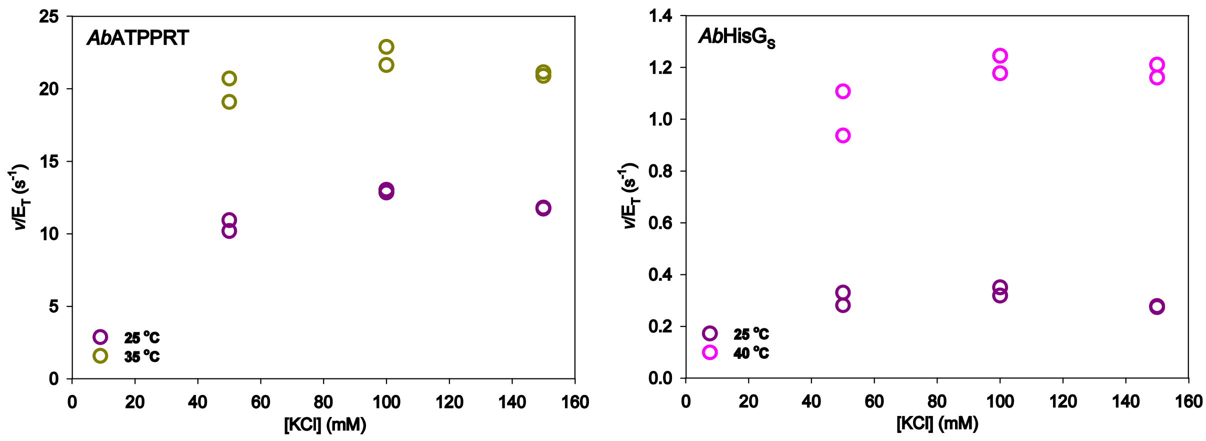

Supplementary Figure 15 Dependence of *Ab*ATPPRT and *Ab*HisG$_S$ activities on KCl concentration at different temperatures. All data points are shown for two independent measurements.

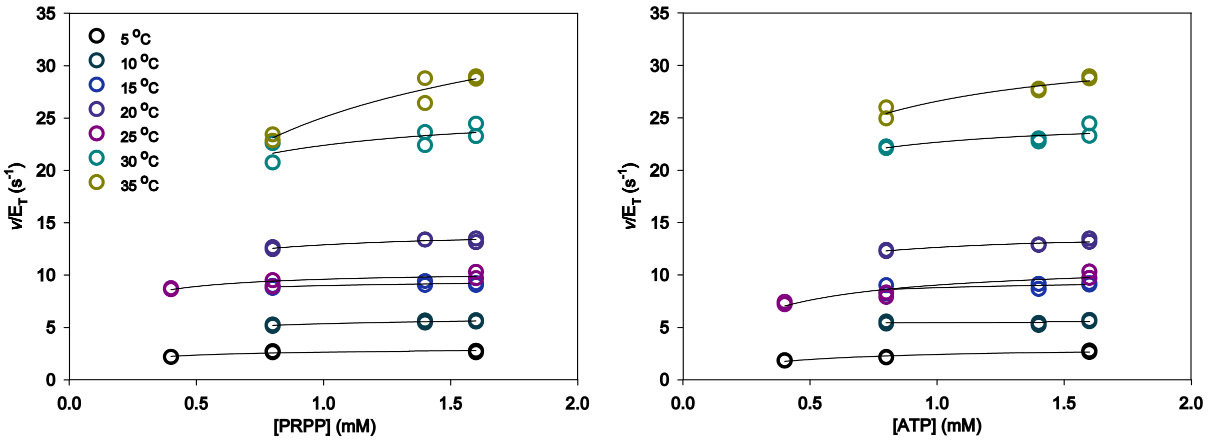

Supplementary Figure 16 Substrate saturation curves for *Ab*ATPPRT at different temperatures. Only concentrations near saturation were used. All data points are shown for two independent measurements, except for curves at 5 °C, where all data points are shown for three independent measurements. Lines are best fit of the data to equation (2).

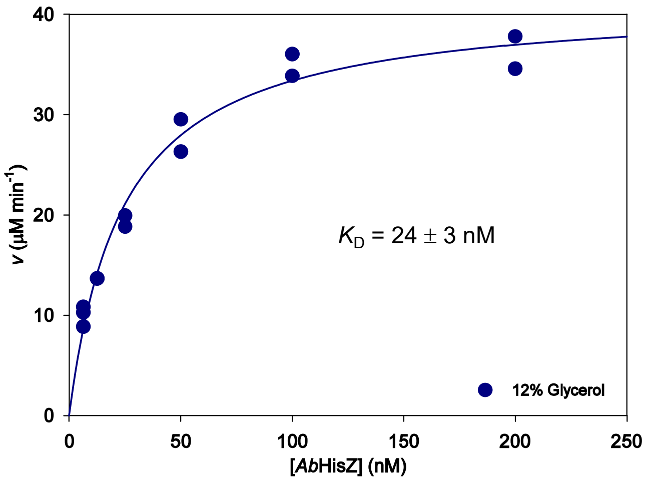

Supplementary Figure 17 Dependence of the rate of reaction catalysed by *Ab*HisGs at 35 °C on the concentration of *Ab*HisZ in the presence of 12% glycerol (v/v). All data points are shown for two independent measurements, except at 6.25 nM, where three independent measurements were performed. Best fit of the data to equation (3) is shown as solid line.

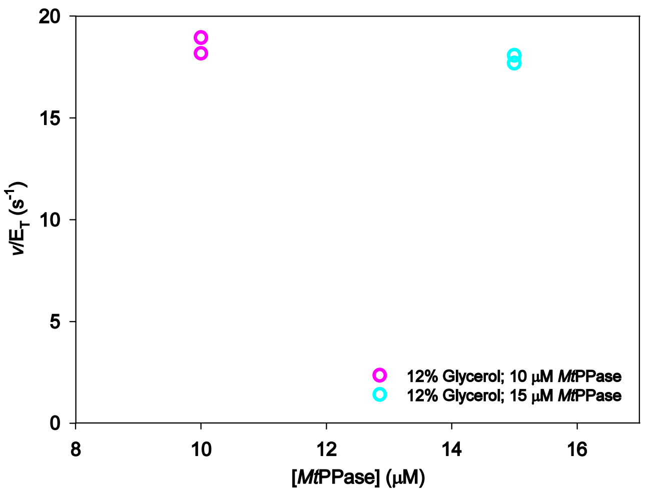

Supplementary Figure 18 Rate of reaction catalysed by *Ab*ATPPRT at 35 °C in the presence of 12% glycerol (v/v) and different concentrations of *Mt*PPase. All data points are shown. Two independent measurements were carried out.

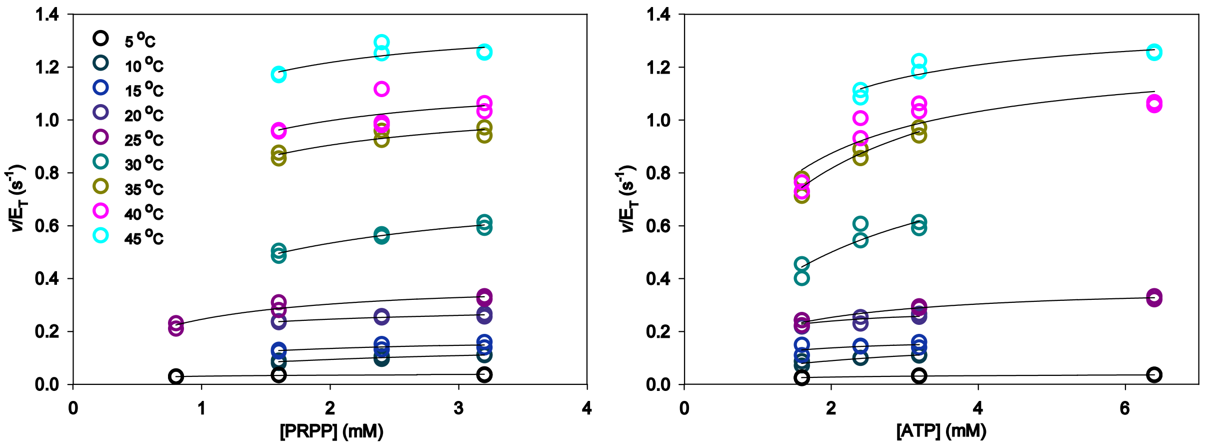

Supplementary Figure 19 Substrate saturation curves for *Ab*HisG$_S$ at different temperatures. Only concentrations near saturation were used. All data points are shown for two independent measurements, except for curves at 5 °C, where all data points are shown for three independent measurements. Lines are best fit of the data to equation (2).

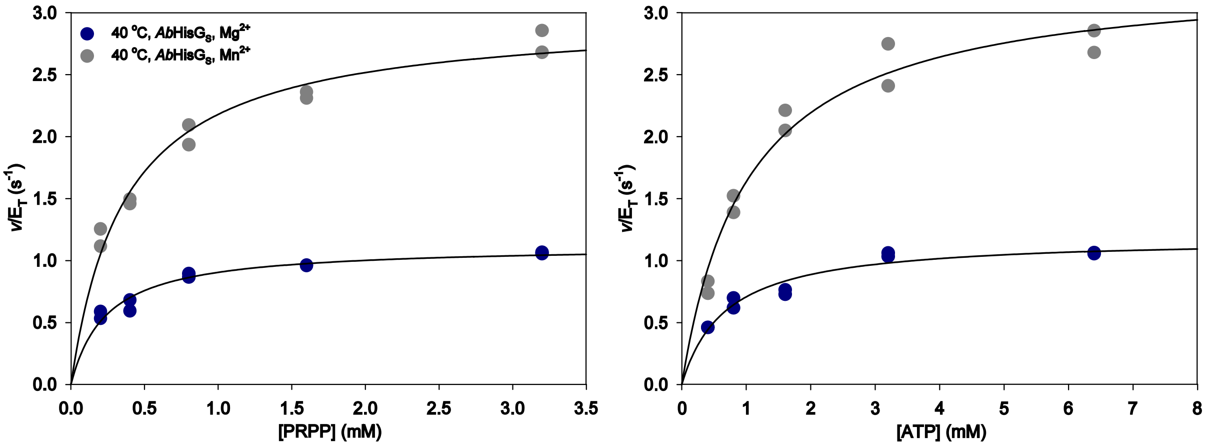

Supplementary Figure 20 Substrate saturation curves for *Ab*HisG$_S$ in the presence of either Mg^{2+} or Mn^{2+} at 40 °C. All data points are shown for two independent measurements. Lines are best fit of the data to equation (2).

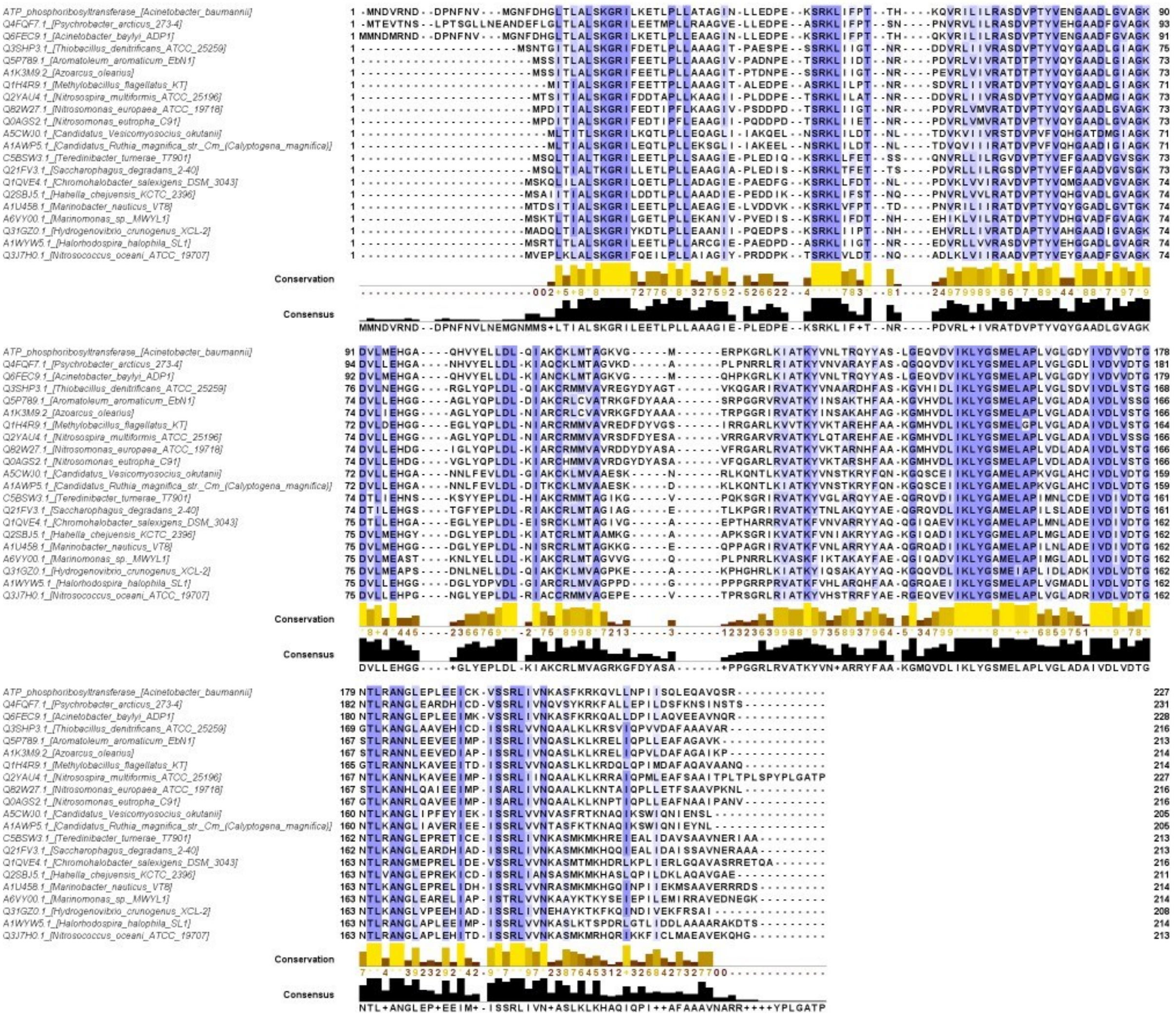

Supplementary Figure 21 Representative multiple sequence alignments of $HisG_S$ orthologues. In total, 205 amino acid sequences were aligned in Clustal Omega. Only the top 20 most conserved sequences in relation to *Ab*$HisG_S$ are shown.

a

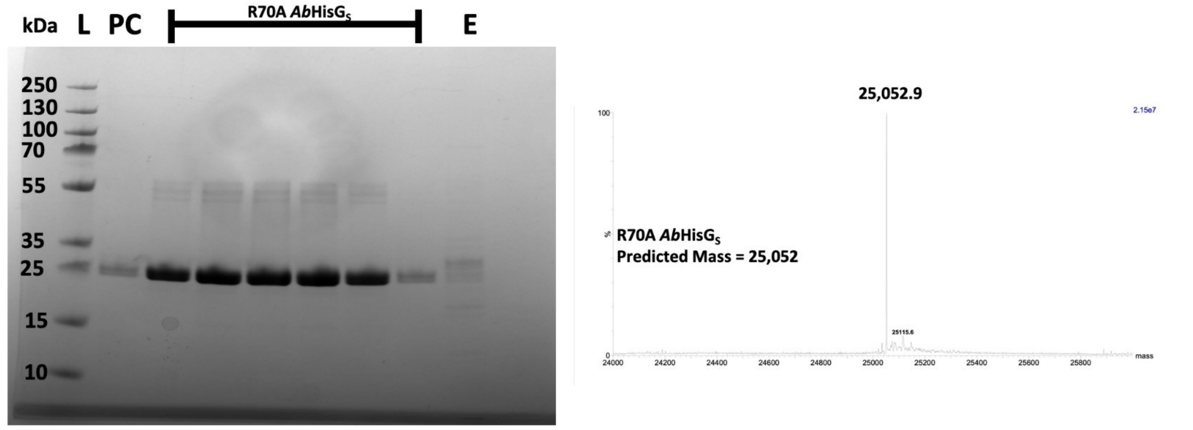

b

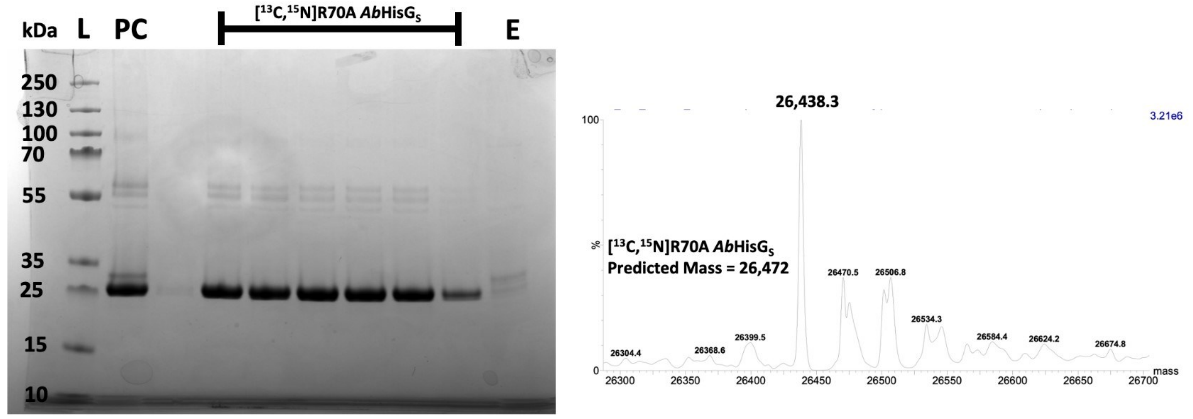

Supplementary Figure 22 SDS-PAGE and ESI/TOF-MS analysis of purified R70A-*Ab*HisG$_S$ isotopologues eluted from the HisTrap FF column in the second chromatography. **a** Unlabelled R70A-*Ab*HisG$_S$. **b** [^{13}C,^{15}N]R70A-*Ab*HisG$_S$. Lanes are as follows: L is the MW marker; PC is the pre-column sample; the top bar encompasses the flowthrough, which was pooled; E is the elution, which was discarded. The MW marker is the PageRuler Plus Prestained. Images **a** and **b** are representative of one batch of the respective *Ab*HisG$_S$ preparation.

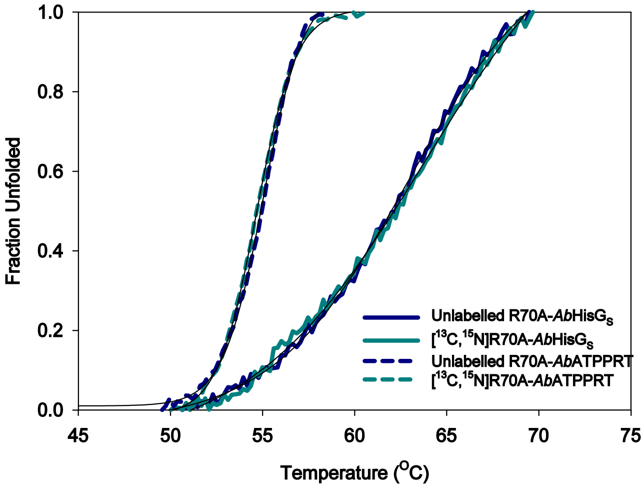

Supplementary Figure 23 DSF-based thermal denaturation of R70A-*Ab*HisG$_S$ and R70A-*Ab*ATPPRT isotopologues. Traces are averages of three independent measurements. Lines of best fit to equation (1) are in grey. T_ms are 62.8 ± 0.1 °C, 63.8 ± 0.2 °C, 55.04 ± 0.03 °C, 54.68 ± 0.01 °C for unlabelled R70A-*Ab*HisG$_S$, unlabelled R70A-*Ab*ATPPRT, [^{13}C,^{15}N]R70A-*Ab*HisG$_S$, [^{13}C,^{15}N]*Ab*ATPPRT, respectively.

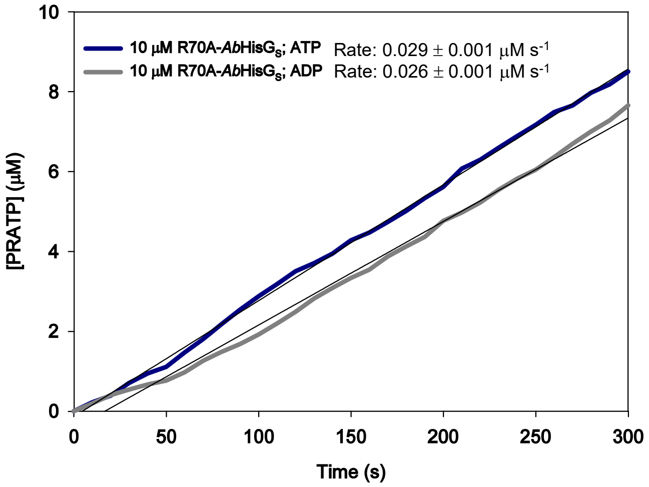

Supplementary Figure 24 Product formation time course of the reaction catalysed by R70A-*Ab*HisG$_S$ at 25 °C with either ATP or ADP as substrate. Traces are average of two independent measurements, and black lines are linear regressions of the data, producing rates as value plus or minus fitting error. In the case of ADP, the product measured is PRADP instead of PRATP.

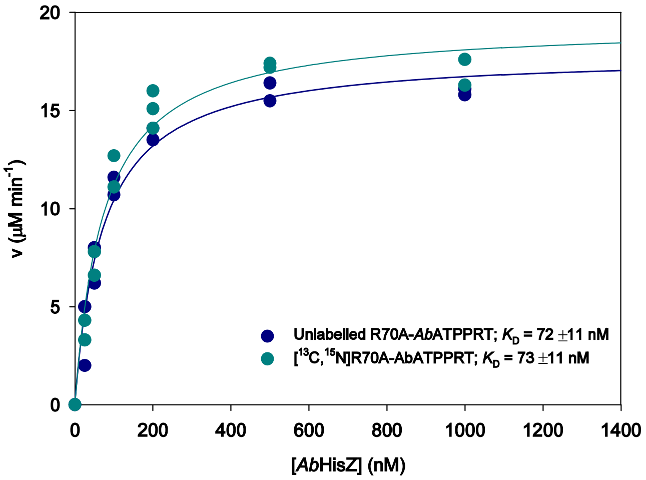

Supplementary Figure 25 Dependence of the rate of reaction catalysed by R70A-*Ab*HisG$_S$ isotopologues at 25 °C on the concentration of *Ab*HisZ. All data points are shown for two independent measurements, except at 200 nM *Ab*HisZ with [^{13}C,^{15}N]R70A-*Ab*HisG$_S$, where three independent measurements were performed. Best fit of the data to equation (3) is shown as solid line.

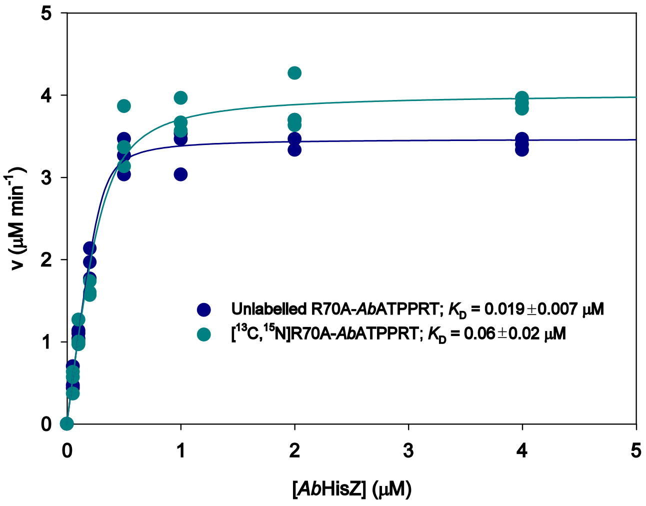

Supplementary Figure 26 Dependence of the rate of reaction catalysed by R70A-*Ab*HisG$_S$ isotopologues at 5 °C on the concentration of *Ab*HisZ. All data points are shown for three independent measurements. Best fit of the data to equation (3) is shown as solid line.

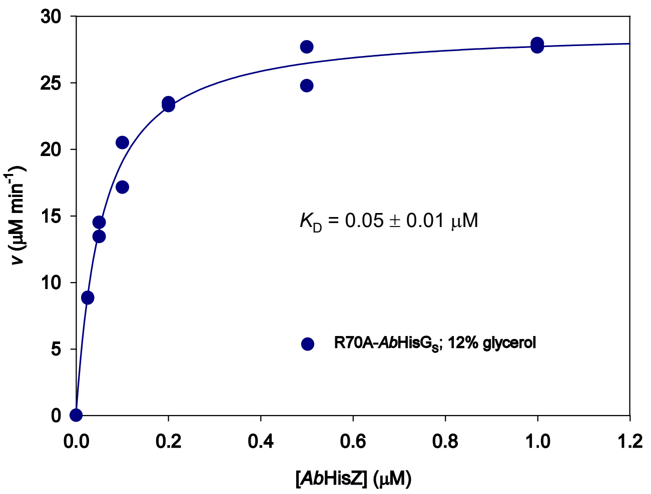

Supplementary Figure 27 Dependence of the rate of reaction catalysed by R70A-*Ab*HisG$_S$ at 25 °C on the concentration of *Ab*HisZ in the presence of 12% glycerol (v/v). All data points are shown for two independent measurements. Best fit of the data to equation (3) is shown as solid line.

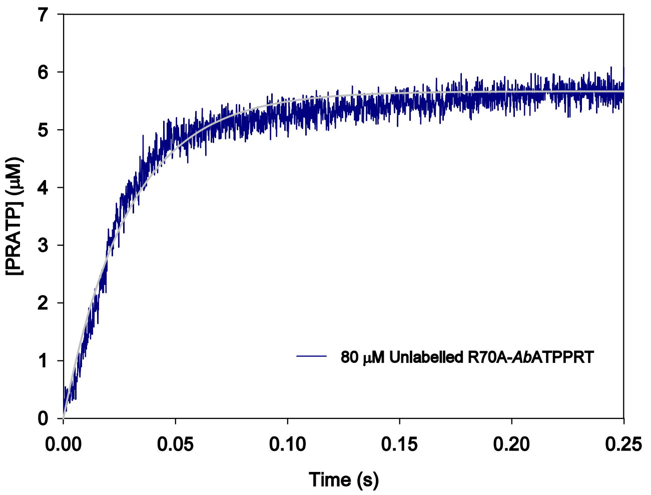

Supplementary Figure 28 Pre-steady-state kinetics of PRATP formation by R70A-*Ab*ATPPRT under single-turnover conditions at 25 °C. Line in colour is the average of six replicates. Thin grey line is best fit of the data to equation (9).

Supplementary Table 1 Apparent steady-state kinetic parameters (mean ± fitting error) at 25 °C for *Ab*ATPPRT with unlabelled *Ab*HisG_S from the M9, M9 high cell density, and LB expression methods.

***Ab*HisGs expression**	**K_M^{PRPP} (mM)**	**K_M^{ATP} (mM)**	**k_{cat} (s^{-1})**	**k_{cat}/K_M^{PRPP} (M^{-1} s^{-1})**	**k_{cat}/K_M^{ATP} (M^{-1} s^{-1})**
LB[a]	0.17 ± 0.01	0.18 ± 0.02	10.5 ± 0.3	(6.1 ± 0.4) × 10^4	(5.8 ± 0.7) × 10^4
M9	0.12 ± 0.01	0.13 ± 0.02	9.6 ± 0.2	(8.0 ± 0.7) × 10^4	(7.4 ± 0.6) × 10^4
M9 high cell density	0.14 ± 0.01	0.19 ± 0.02	10.9 ± 0.3	(7.8 ± 0.6) × 10^4	(5.7 ± 0.6) × 10^4

[a]Previously published values.

Supplementary Table 2 Apparent steady-state kinetic parameters (mean ± fitting error) at 25 °C for *Ab*HisGs isotopologues.

***Ab*HisGs isotopologue**	**K_M^{PRPP} (mM)**	**K_M^{ATP} (mM)**	**k_{cat} (s^{-1})**	**k_{cat}/K_M^{PRPP} (M^{-1} s^{-1})**	**k_{cat}/K_M^{ATP} (M^{-1} s^{-1})**
**Unlabelled**	0.61 ± 0.06	0.83 ± 0.06	0.38 ± 0.01	630 ± 60	460 ± 30
**^{15}N**	0.54 ± 0.04	0.9 ± 0.1	0.38 ± 0.01	700 ± 70	420 ± 50
**^{13}C,^{15}N**	0.33 ± 0.01	0.8 ± 0.1	0.34 ± 0.01	1020 ± 40	420 ± 50
**^{2}H,^{13}C,^{15}N**	0.42 ± 0.02	0.59 ± 0.03	0.34 ± 0.04	810 ± 40	580 ± 30

Supplementary Table 3 Apparent K_D (mean ± fitting error) for *Ab*HisZ with *Ab*HisG$_S$ isotopologues.

***Ab*ATPPRT isotopologue**	**K_D (nM) at 25 °C**	**K_D (nM) at 5 °C**
**Unlabelled**	16 ± 3	41 ± 5
**^{15}N**	23 ± 4	95 ± 12
**^{13}C,^{15}N**	11 ± 2	10 ± 2
**^{2}H,^{15}N**	17 ± 2	94 ± 10
**^{2}H,^{13}C,^{15}N**	12 ± 2	24 ± 4

Supplementary Table 4 Apparent steady-state kinetic parameters (mean ± fitting error) at 25 °C for *Ab*ATPPRT isotopologues.

***Ab*ATPPRT isotopologue**	**K_M^{PRPP} (mM)**	**K_M^{ATP} (mM)**	**k_{cat} (s^{-1})**	**k_{cat}/K_M^{PRPP} (M^{-1} s^{-1})**	**k_{cat}/K_M^{ATP} (M^{-1} s^{-1})**
**Unlabelled**	0.14 ± 0.01	0.19 ± 0.02	10.9 ± 0.3	(7.8 ± 0.6) × 10^4	(5.7 ± 0.6) × 10^4
**^{15}N**	0.18 ± 0.03	0.17 ± 0.03	10.8 ± 0.4	(6 ± 1) × 10^4	(6 ± 1) × 10^4
**^{13}C,^{15}N**	0.106 ± 0.009	0.19 ± 0.02	10.3 ± 0.2	(9.7 ± 0.8) × 10^4	(5.4 ± 0.6) × 10^4
**^{2}H,^{15}N**	0.17 ± 0.02	0.19 ± 0.03	10.1 ± 0.3	(5.9 ± 0.7) × 10^4	(5.3 ± 0.9) × 10^4
**^{2}H,^{13}C,^{15}N**	0.15 ± 0.02	0.21 ± 0.03	10.6 ± 0.3	(7 ± 1) × 10^4	(5.0 ± 0.7) × 10^4

Supplementary Table 5 *Ab*ATPPRT isotopologues allosteric inhibition parameters (mean ± fitting error) for histidine at 25 °C.

*Ab*ATPPRT isotopologue	IC_{50} (μM)	*h*
Unlabelled	93 ± 6	1.4 ± 0.1
^{15}N	110 ± 11	1.4 ± 0.2
$^{13}C,^{15}N$	113 ± 5	1.7 ± 0.1
$^{2}H,^{15}N$	113 ± 8	1.5 ± 0.2
$^{2}H,^{13}C,^{15}N$	72 ± 2	1.28 ± 0.04

Supplementary Table 6 Apparent steady-state kinetic parameters and $^{HE}k_{cat}$ (mean ± fitting error) at 5 °C for *Ab*HisG$_S$ isotopologues.

*Ab*HisG$_S$ isotopologue	K_M^{PRPP} (mM)	K_M^{ATP} (mM)	k_{cat} (s^{-1})	k_{cat}/K_M^{PRPP} (M^{-1} s^{-1})	k_{cat}/K_M^{ATP} (M^{-1} s^{-1})	$^{HE}k_{cat}$
Unlabelled	0.34 ± 0.03	1.07 ± 0.06	0.041 ± 0.001	120 ± 10	39 ± 2	1.00 ± 0.03
^{15}N	0.35 ± 0.01	0.89 ± 0.09	0.040 ± 0.001	116 ± 4	46 ± 5	1.02 ± 0.04
$^{13}C,^{15}N$	0.25 ± 0.02	1.0 ± 0.1	0.043 ± 0.001	170 ± 10	43 ± 4	0.95 ± 0.03
$^{2}H,^{13}C,^{15}N$	0.27 ± 0.02	1.13 ± 0.07	0.039 ± 0.001	140 ± 10	34 ± 2	1.05 ± 0.04

Supplementary Table 7 Single-turnover rate constants (mean ± fitting error) with *Ab*ATPPRT isotopologues at 5 °C.

*Ab*ATPPRT isotopologue	k_2	k_3
**Unlabelled (1st prep)**	94 ± 2	51.0 ± 0.5
**Unlabelled (2nd prep)**	113 ± 2	59.0 ± 0.7
**$^{13}C,^{15}N$**	73 ± 2	48.1 ± 0.8
**$^{2}H,^{13}C,^{15}N$**	116 ± 2	52.3 ± 0.3

Supplementary Table 8 DSF-based T_m (mean ± fitting error) for *Ab*ATPPRT isotopologues in the presence and absence of PRATP.

*Ab*ATPPRT isotopologue	(−PRATP) T_m (°C)	(+PRATP) T_m (°C)	ΔT_m (°C)
**Unlabelled**	55.44 ± 0.02	56.73 ± 0.04	+1.29 ± 0.04
**$^{13}C,^{15}N$**	55.33 ± 0.02	55.80 ± 0.03	+0.47 ± 0.02
**$^{2}H,^{13}C,^{15}N$**	55.32 ± 0.01	56.42 ± 0.01	+1.10 ± 0.01

Supplementary Table 9 Apparent steady-state kinetic parameters (mean ± fitting error) at 25 °C for *Ab*ATPPRT isotopologues with ADP as substrate.

***Ab*ATPPRT isotopologue**	**K_M^{PRPP} (mM)**	**K_M^{ADP} (mM)**	**k_{cat} (s^{-1})**	**k_{cat}/K_M^{PRPP} (M^{-1} s^{-1})**	**k_{cat}/K_M^{ADP} (M^{-1} s^{-1})**	**$^{HE}k_{cat}$**
**Unlabelled**	0.096 ± 0.005	0.35 ± 0.04	16.6 ± 0.4	(1.73 ± 0.1) × 10^5	(4.7 ± 0.6) × 10^4	1.00 ± 0.04
**^{2}H,^{15}N**	0.08 ± 0.01	0.30 ± 0.06	15.1 ± 0.5*	(1.9 ± 0.2) × 10^5	(5 ± 1) × 10^4	1.10 ± 0.05
**^{2}H,^{13}C,^{15}N**	0.085 ± 0.008	0.28 ± 0.05	14.6 ± 0.4**	(1.7 ± 0.2) × 10^5	(5.2 ± 0.2) × 10^4	1.14 ± 0.04

*$p > 0.14$ and **$p > 0.07$ by a Student's *t*-test in comparison with unlabelled *Ab*ATPPRT k_{cat}.

Supplementary Table 10 Apparent steady-state kinetic parameters (mean ± fitting error) at 5 °C for *Ab*ATPPRT isotopologues with ADP as substrate.

***Ab*ATPPRT isotopologue**	**K_M^{PRPP} (mM)**	**K_M^{ADP} (mM)**	**k_{cat} (s^{-1})**	**k_{cat}/K_M^{PRPP} (M^{-1} s^{-1})**	**k_{cat}/K_M^{ADP} (M^{-1} s^{-1})**	**$^{HE}k_{cat}$**
**Unlabelled**	0.079 ± 0.005	0.28 ± 0.04	3.1 ± 0.1	(3.9 ± 0.3) × 10^4	(1.1 ± 0.2) × 10^4	1.00 ± 0.05
**^{2}H,^{15}N**	0.14 ± 0.01	0.28 ± 0.04	3.12 ± 0.06	(2.2 ± 0.2) × 10^4	(1.1 ± 0.2) × 10^4	0.99 ± 0.04
**^{2}H,^{13}C,^{15}N**	0.119 ± 0.007	0.27 ± 0.04	3.4 ± 0.1	(2.9 ± 0.2) × 10^4	(1.3 ± 0.2) × 10^4	0.91 ± 0.04

Supplementary Table 11 Apparent steady-state kinetic parameters (mean ± fitting error) at 25 °C for unlabelled R70A-*Ab*ATPPRT with either ATP or ADP as substrate.

**Nucleotide**	**K_M^{PRPP} (mM)**	**$K_M^{AXP^a}$ (mM)**	**k_{cat} (s^{-1})**	**k_{cat}/K_M^{PRPP} (M^{-1} s^{-1})**	**$k_{cat}/K_M^{AXP^a}$ (M^{-1} s^{-1})**
**ATP**	0.30 ± 0.04	0.7 ± 0.1	2.17 ± 0.06	(7 ± 1) × 10^3	(3.1 ± 0.5) × 10^3
**ADP**	0.50 ± 0.05	1.3 ± 0.1	2.47 ± 0.06	(4.9 ± 0.5) × 10^3	(1.9 ± 0.2) × 10^3

^{a}X denotes either T (for ATP) or D (for ADP).

Supplementary Table 12 Apparent steady-state kinetic parameters (mean ± fitting error) at 5 °C for R70A-*Ab*ATPPRT isotopologues.

***Ab*ATPPRT isotopologue**	**K_M^{PRPP} (mM)**	**K_M^{ATP} (mM)**	**k_{cat} (s^{-1})**	**k_{cat}/K_M^{PRPP} (M^{-1} s^{-1})**	**k_{cat}/K_M^{ATP} (M^{-1} s^{-1})**
**Unlabelled**	0.32 ± 0.03	0.79 ± 0.06	0.284 ± 0.005	(8.9 ± 0.8) × 10^2	(3.6 ± 0.3) × 10^2
**$^{13}C,^{15}N$**	0.18 ± 0.01	0.75 ± 0.09	0.267 ± 0.005*	(1.48 ± 0.09) × 10^3	(3.6 ± 0.4) × 10^2

*$p > 0.14$ by a Student's *t*-test in comparison with unlabelled *Ab*ATPPRT k_{cat}.

Supplementary Table 13 Apparent steady-state kinetic parameters (mean ± fitting error) at 25 °C for R70A-*Ab*ATPPRT isotopologues.

***Ab*ATPPRT isotopologue**	**K_M^{PRPP} (mM)**	**K_M^{ATP} (mM)**	**k_{cat} (s^{-1})**	**k_{cat}/K_M^{PRPP} (M^{-1} s^{-1})**	**k_{cat}/K_M^{ATP} (M^{-1} s^{-1})**
**Unlabelled**	0.30 ± 0.04	0.7 ± 0.1	2.17 ± 0.06	(7 ± 1) × 10^3	(3.1 ± 0.5) × 10^3
**$^{13}C,^{15}N$**	0.38 ± 0.03	0.82 ± 0.09	2.01 ± 0.04*	(5.3 ± 0.4) × 10^3	(2.5 ± 0.3) × 10^3

*$p > 0.15$ by a Student's *t*-test in comparison with unlabelled *Ab*ATPPRT k_{cat}.

Supplementary Table 14 Differential M9 minimum medium supplementation for production of WT- and R70A-*Ab*HisG$_S$ isotopologues.

Labelling	Nitrogen Source	Carbon Source	Solvent
**Natural isotope abundance**	NH_4Cl	D-glucose	H_2O
**^{15}N**	[^{15}N]NH_4Cl	D-glucose	H_2O
**^{15}N,^{13}C**	[^{15}N]NH_4Cl	[$^{13}C_6$]D-glucose	H_2O
**^{2}H,^{15}N**	[^{15}N]NH_4Cl	[$^{2}H_7$]D-glucose	D_2O
**^{2}H,^{13}C,^{15}N**	[^{15}N]NH_4Cl	[$^{13}C_6$,$^{2}H_7$]D-glucose	D_2O
